# Comparative analysis of hypobaric versus hyperbaric bupivacaine for spinal anesthesia in day-case anorectal surgery: A prospective observational study

**DOI:** 10.1097/MD.0000000000041904

**Published:** 2025-03-21

**Authors:** Kudret Yasemin Yalniz, Semih Baskan

**Affiliations:** a Department of Anesthesiology and Reanimation, Kahramankazan Hamdi Eris Public Hospital, Ankara, Turkey; b Department of Anesthesiology and Reanimation, Ankara Bilkent City Hospital, Ankara Yildirim Beyazit University, Ankara, Turkey.

**Keywords:** anorectal surgery, day-case surgery, hyperbaric bupivacaine, hypobaric bupivacaine, spinal anesthesia

## Abstract

**Background:**

This study aimed to compare the efficacy, safety, and discharge times of patients undergoing day-case anorectal surgery using 5 mg hyperbaric bupivacaine versus 5 mg hypobaric bupivacaine. The evaluation was based on Fast-Track Scoring criteria, which assess the suitability of patients for day-case surgery.

**Methods:**

A prospective observational study was conducted at Ankara City Hospital, including 80 patients aged 18 to 65 years within American Society of Anesthesiologists I–II risk groups scheduled for day-case anorectal surgery. All patients received spinal anesthesia with either 5 mg of 0.5% hyperbaric bupivacaine or 5 mg of 0.5% hypobaric bupivacaine. Hemodynamic parameters, sensory and motor block durations, time to first analgesic need, urination time, and discharge time were meticulously recorded and analyzed. Statistical analyses were performed using the independent samples *t* test and Mann–Whitney *U* test, with significance set at α = 0.05.

**Results:**

The hypobaric group exhibited significantly shorter sensory and motor block durations, enabling faster surgical readiness and earlier discharge. Both groups maintained comparable hemodynamic stability and postoperative complication rates. Shorter discharge times in the hypobaric group may offer potential benefits for patient turnover and cost-effectiveness.

**Conclusion:**

For patients undergoing day-case anorectal surgery, 5 mg hypobaric bupivacaine provides faster recovery and shorter discharge time than hyperbaric bupivacaine, without compromising safety. These findings suggest that hypobaric bupivacaine may be a more suitable choice in day-case settings, contributing to improved resource utilization and patient satisfaction.

## 1. Introduction

Anorectal diseases, such as hemorrhoids, anal polyps, pilonidal sinus, anal fissures, anal fistulas, and rectoceles, are common. Surgical treatment for these conditions can be performed using various anesthetic methods, including general anesthesia, neuraxial anesthesia, local anesthetic infiltration, and combined anesthesia techniques.^[1,2]^

In recent years, more than 90% of anorectal surgeries have been planned as day-case procedures due to their cost-effectiveness and safety. Day-case surgery is increasingly preferred worldwide as it significantly reduces healthcare costs and is safe. Quick recovery from the chosen anesthesia method enhances both surgeon and patient satisfaction. Consequently, there is an increasing trend toward regional anesthesia techniques, which offer several advantages over general anesthesia.^[3]^

Anesthesia-related complications and side effects can affect discharge times in day-case patients, making the choice of anesthesia method and agents critical. Regional anesthesia applications allow patients to remain conscious during the procedure, maintain spontaneous respiration, and preserve airway reflexes, thus providing postoperative analgesia and offering advantages over general anesthesia in appropriate cases.^[4]^

Recent studies have highlighted the effectiveness of various regional anesthesia techniques, including the use of different baricities of bupivacaine in spinal anesthesia for anorectal surgery.^[5–10]^ Pharmacological differences between hypobaric and hyperbaric bupivacaine influence their preference in spinal anesthesia applications. Hyperbaric bupivacaine shows a pronounced distribution in the cerebrospinal fluid under the effect of gravity due to its density, whereas hypobaric bupivacaine provides a more homogeneous distribution.^[5,7]^ The primary aim of this study is to compare the efficacy and recovery profiles of 5 mg hyperbaric and 5 mg hypobaric bupivacaine in patients undergoing day-case anorectal surgery and evaluate their suitability for day-case surgery based on Fast-Track Scoring criteria.

## 2. Materials and methods

### 2.1. Study design and approval

This prospective observational study was conducted at Ankara City Hospital following the approval of the Ethics Committee (Decision No: E1-22-2335, Date: January 26, 2022). Written informed consent was obtained from all patients. The study included 80 patients aged 18 to 65 years in American Society of Anesthesiologists (ASA) I–II risk groups undergoing day-case anorectal surgery under spinal anesthesia.

- Inclusion criteria: Patients aged 18 to 65 years in ASA I–II risk groups, scheduled for day-case anorectal surgery.
- Exclusion criteria: Known hypersensitivity to amide-type local anesthetics, refusal of regional anesthesia, preoperative motor and sensory deficits, bleeding diathesis, and infections that contraindicate spinal anesthesia.

### 2.2. Preoperative preparation

An 18 G intravenous cannula was inserted into the nondominant hand of each patient before spinal anesthesia. Standard monitoring included electrocardiography, oxygen saturation (SpO2), and noninvasive blood pressure measurements. Baseline hemodynamic parameters, including heart rate and blood pressure, were recorded.

### 2.3. Anesthesia procedure

Spinal anesthesia was administered in the sitting position at the L4-5 or L5-S1 interspinous space using a 25 G Quincke spinal needle. Patients were randomly assigned to 2 groups: Group 1 received 5 mg of 0.5% hyperbaric bupivacaine (Marcain® spinal heavy 0.5%, AstraZeneca), and Group 2 received 5 mg of 0.5% isobaric bupivacaine (Marcain® 0.5%, AstraZeneca) diluted with 1 mL of sterile distilled water to create hypobaric bupivacaine, which was administered intrathecally over 30 seconds. The time of drug administration completion was considered “0 minute” Patients in the hyperbaric group were kept in a sitting position for saddle block until achieving the appropriate dermatomal block, while patients in the hypobaric group were placed in the Trendelenburg position. After achieving an adequate dermatomal block, patients were placed in the surgical positions of prone, jack-knife, or lithotomy. Sensory block level, motor block degree, and hemodynamic parameters such as heart rate, systolic arterial pressure, diastolic arterial pressure, mean arterial pressure, and SpO2 were recorded every 5 minutes until the end of surgery. Sensory block level was assessed using a “pin prick” test with a blunt needle on the corresponding dermatomes according to the standard dermatome map, and motor block level was evaluated using the Modified Bromage Scale (0 = no paralysis, able to lift thigh, leg, and foot; 1 = unable to move thigh, able to move knee; 2 = unable to move knee, able to move ankle; 3 = unable to move any part of the lower limb). After confirming the block in all appropriate dermatomes for surgery using the “pin prick” test performed every minute, the operation was allowed to begin. The time from the puncture to the start of surgery was recorded. Analgesia in the surgical area as confirmed by the “pin prick” test was considered sufficient for sensory block. Sensory and motor block assessments were performed during the operation in collaboration with the surgical team to avoid interfering with the procedure.

### 2.4. Sensory block assessments

Sensory block assessments were performed and recorded according to the following definitions:

- Time to achieve adequate anesthesia for surgery: The time from spinal anesthesia administration until the “pin prick” test indicated the absence of sharp sensation in all dermatomes appropriate for the surgical area.
- Time to reach maximum level: The time until the highest dermatomal level of sensory block was achieved, as assessed by the “pin prick” test.
- Maximum level reached: The highest dermatomal level achieved where the sharp sensation was absent, as assessed by the “pin prick” test.
- Time to reach S4 dermatomal level of sensory block: The time until the absence of sharp sensation at the S4 dermatome, as assessed by the “pin prick” test.
- Time of sensory block resolution: The time from the initiation of the sensory block until the sensory block resolved completely in all affected dermatomes, as determined by the “pin prick” test.

### 2.5. Motor block assessments

Motor block assessments were performed and recorded according to the following definitions:

- Maximum motor block degree: The highest modified Bromage score achieved at specified time intervals.
- Time to achieve maximum motor block degree: The time from the administration of spinal anesthesia until the maximum motor block degree was obtained.
- Time of motor block resolution: The time from the onset of motor block until the modified Bromage score returned to 0.

### 2.6. Monitoring and measurements

Hemodynamic parameters, motor block degree, and sensory block level were measured every 5 minutes until the procedure was completed. Motor block was evaluated using the Modified Bromage Scale. Complications such as bradycardia, hypotension, nausea, vomiting, agitation, shivering, and respiratory depression were observed. In the day surgery unit, patients were monitored for vital signs, sensory block regression, and discharge criteria postsurgery. Patient satisfaction, time to first urination, and discharge times were recorded.

During surgery, patients were monitored for side effects such as hypotension, bradycardia, nausea, vomiting, pain, shivering, agitation, and respiratory depression, and any complications observed were recorded. Total surgery time was also documented. Patients were contacted 24 to 30 hours postoperatively to assess for nausea, vomiting, headache, and anesthesia satisfaction (1 = not satisfied, 2 = somewhat satisfied, 3 = satisfied, 4 = very satisfied). If necessary, patients were called back to the hospital for treatment, which was documented. Any intraoperative sedation requirements and medications administered were recorded on the follow-up form.

After the surgical procedure, patients were placed in a horizontal position in the day surgery unit. In the day surgery unit, monitoring of heart rate, systolic arterial pressure, diastolic arterial pressure, mean arterial pressure, and SpO2 continued every 15 minutes. Monitoring of the Modified Bromage Scale and dermatome sensory levels was also conducted and recorded.

In the day surgery unit, the time to first urination, mobilization times, and the complete resolution of both sensory and motor blocks were monitored and recorded as part of the discharge criteria. Patients were evaluated according to the Fast-Track scoring system (Table 1), with discharge planned for those who achieved a score of 12 out of 14 and demonstrated full recovery of motor function.

**Table 1:** Fast-track scoring system.

Criteria	Score
Vital signs stability	
Blood pressure within 20% of baseline	2
Blood pressure deviates slightly (within 20–40% of baseline)	1
Blood pressure deviates significantly (>40% of baseline)	0
Activity and mobility	
Able to stand and walk independently	2
Able to stand but requires assistance to walk	1
Unable to stand or walk	0
Nausea and vomiting	
No nausea or vomiting	2
Mild nausea controlled with medication	1
Severe nausea or vomiting uncontrolled with medication	0
Pain control	
Minimal pain controlled without medication	2
Moderate pain controlled with medication	1
Severe pain uncontrolled with medication	0
Hydration and oral intake	
Able to drink fluids without nausea	2
Able to take small sips only	1
Unable to take fluids	0
Bladder function	
Has voided independently	2
Has not voided but reports no discomfort	1
Has not voided and reports discomfort	0

### 2.7. Statistical analysis

Data analysis was performed using IBM Statistical Package for the Social Sciences 25.0 (IBM Corp., Armonk). Descriptive statistics (frequency, percentage, mean, standard deviation, median, and min-max) were used to summarize the data. For qualitative data, the chi-square (χ^2^) test was used. Normal distribution of quantitative data was assessed using Kolmogorov–Smirnov and Shapiro–Wilk tests, skewness-kurtosis, and graphical methods (histogram, Q–Q plot, stem and leaf, boxplot).

For normally distributed quantitative data, the independent samples *t* test or 1-way analysis of variance was used for group comparisons, and the paired *t* test or repeated measures analysis of variance was used for repeated measures. For non-normally distributed data, the Mann–Whitney *U* test or Kruskal–Wallis test was used for group comparisons and the Wilcoxon test or Friedman test for repeated measures. Relationships between variables were evaluated using Pearson’s or Spearman’s correlation test. Statistical significance was set at *P* = .05.

## 3. Results

### 3.1. Demographic and baseline characteristics

In the comparison of demographic characteristics, including gender, age, height, weight, BMI, and ASA classification, there were no statistically significant differences between the groups (Table 2).

**Table 2:** Comparison of patient characteristics between groups.

Characteristic	Group I, n = 40	Group II, n = 40	*P* value
Sex (F/M)	9/31	9/31	1.000*
Age (yr)	40.7 ± 14.2	40.3 ± 12.0	.899†
Height (cm)	172.5 ± 7.2	170.6 ± 9.3	.296†
Weight (kg)	82.7 ± 16.0	82.7 ± 14.8	.983†
BMI (kg/m²)	27.7 ± 4.6	28.5 ± 5.3	.461†
ASA (I/II)	14/26	22/18	.116*

Data are expressed as n (%) or average ± standard deviation.

ASA = American Society of Anesthesiologists physical status classification, BMI = body mass index, F = female, Group I = hyperbaric bupivacaine, Group II = hipobaric bupivacaine, M = male, n (%) = number of patients (percentage), SD = standard deviation.

* Chi-square test.

† Independent samples *t* test.

### 3.2. Surgery and anesthesia times

There was a statistically significant difference in the duration of surgery between the 2 groups, with Group I (hyperbaric bupivacaine) having longer surgery times (Table 3). The difference in surgical duration could be attributed to the type of procedures performed, specifically pilonidal sinus excision, which was more frequent in Group I. Pilonidal sinus excision is a technically complex and time-consuming procedure, involving tissue removal and wound closure, often leading to extended surgical times.

**Table 3:** Comparison of surgery and anesthesia times.

Parameter	Group I, n = 40	Group II, n = 40	*P* value
Surgery type			
Anal fistulectomy	22 (55.0%)	25 (62.5%)	–
Pilonidal sinus excision	9 (22.5%)	5 (12.5%)	
Hemorrhoidectomy	4 (10.0%)	7 (17.5%)	
Other	5 (12.5%)	3 (7.5%)	
Surgery duration (min)	25.8 ± 12.0	18.8 ± 9.9	.006
Time to surgical readiness (min)	5.2 ± 1.4	7.3 ± 2.2	.000

Values are presented as number (percentage) or mean ± standard deviation. *P* values are derived from the independent samples *t* test.

Group I = hyperbaric bupivacaine, Group II = hipobaric bupivacaine, n (%) = number of patients (percentage).

A statistically significant difference was found between the groups in terms of the time required to achieve the S4 sensory dermatome block and the time for the sensory block to regress (*P* < .05). Group II (hypobaric bupivacaine) showed a longer duration for S4 dermatome sensory block onset but a shorter time for sensory block regression, as indicated in Table 4.

**Table 4:** Postoperative outcomes.

Parameter	Group I, n = 40	Group II, n = 40	*P* value
*S4 sensory block duration (min*)	3.4 ± 1.5	5.3 ± 1.6	<.001
Time to first analgesic need (h)	4.34 ± 1.72	5.35 ± 2.35	.073
Time to urination (h)	3.71 ± 1.35	3.40 ± 1.19	.277
Time to mobilization (h)	2.19 ± 1.00	1.85 ± 0.75	.093
*Discharge time (h*)	4.23 ± 1.04	3.38 ± 0.77	.000
Patient satisfaction (1–10 scale)	8.2 ± 1.4	9.1 ± 1.0	.001
Willingness to undergo again (%)	90%	95%	.500
Postoperative complications	7 (17.5%)	4 (10.0%)	.516
Nausea and vomiting	*1 (2.5%*)	*3 (7.5%*)	.615
Headache	*2 (5.0%*)	*1 (2.5%*)	1.000
Urinary retention	*2 (5.0%*)	*–*	.494
Hypotension	*1 (2.5%*)	*–*	1.000
Lower back pain	*1 (2.5%*)	*–*	1.000

Values are presented as mean ± standard deviation or number (percentage). *P* values are derived from the independent samples *t* test or chi-square test.

n (%) = number of patients (percentage).

**Figure 1.**
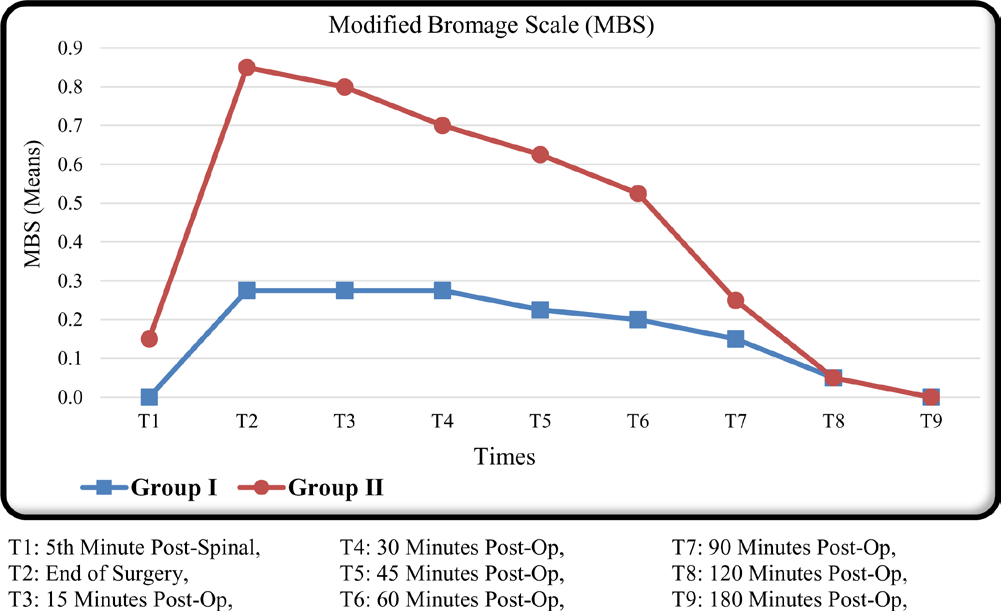
Modified Bromage Scale (MBS; range from 0 to 3). The time to reach an MBS score of 0 was 1.88 ± 0.79 hours in Group I and 1.64 ± 0.59 hours in Group II, with no statistically significant difference between the groups (*P* = .404). MBS = Modified Bromage Scale.

Additionally, comparisons between the groups revealed a statistically significant difference in Modified Bromage Scale scores across all measured time points (*P* < .05). Group II patients consistently had higher Modified Bromage Scale scores, indicating a more pronounced motor block (Fig. 1).

Group II (hypobaric bupivacaine) showed a longer time to surgical readiness after spinal anesthesia than Group I (hyperbaric bupivacaine; Table 3).

## 4. Perioperative outcomes

There was no statistically significant difference in sedation requirements between the groups (*P* > .05). For patients requiring sedation, 0.03 mg/kg midazolam and 1 mcg/kg fentanyl were administered. Sedation was required in 7.5% of patients in Group I (n = 3) and 5% of patients in Group II (n = 2).

Postoperative complications showed no statistically significant difference between the groups (*P* > .05). In Group I, 5 patients (12.5%) experienced complications, compared to 4 patients (10%) in Group II. The observed complications included headache, nausea and vomiting, hypotension, and lower back pain.

There were significant differences in postoperative outcomes, including sensory and motor block characteristics (Fig. 1) discharge time, and patient satisfaction. There was no statistically significant difference between the groups in terms of time to first analgesic requirement, urination time, and mobilization time (*P* > .05; Table 4).

## 5. Discussion

Our statistical analysis revealed that the hypobaric bupivacaine group exhibited higher early-stage Modified Bromage Scale (MBS) scores. This outcome is attributed to the broader dermatome involvement associated with hypobaric bupivacaine and the assessment of MBS at the extremity level. However, as demonstrated in the Figure 1, although the hypobaric group had higher initial MBS scores, the time to reach an MBS score of zero was no statistically significant difference between the groups (*P* = .404). This finding indicates that while the early motor block intensity appeared greater in the hypobaric group, the overall motor block resolution time was similar between the 2 groups, as reflected by the comparable times to achieve complete motor function recovery.

According to the statistical data from our study, while there was no significant difference between groups regarding mobilization and urination times following spinal anesthesia, it was observed that patients receiving hypobaric bupivacaine required additional time to achieve sensory block at the S4 dermatome. However, these patients also experienced a faster resolution of sensory block, resulting in shorter discharge times. Evaluation of hemodynamic data during intraoperative and postoperative monitoring revealed no significant differences between the 2 groups, suggesting comparable hemodynamic stability with both anesthetic techniques.

Despite a clinically negligible delay in sensory block onset and duration, as well as a slight extension in surgical readiness time, the hypobaric bupivacaine group demonstrated faster block resolution and shorter discharge times.

In our study, the rapid recovery profile of hypobaric bupivacaine demonstrated shorter discharge times compared to hyperbaric bupivacaine. This observation is consistent with findings from previous studies. Kaya et al^[11]^ evaluated the hemodynamic and recovery characteristics of hypobaric versus hyperbaric bupivacaine and found similar hemodynamic stability between the 2 formulations. However, the hypobaric solution exhibited faster regression of both sensory and motor blocks, making it particularly suitable for outpatient procedures where rapid recovery is prioritized. Imbelloni et al^[12]^ conducted a comparative study examining the effects of hypobaric and hyperbaric bupivacaine in patients undergoing anorectal surgery in the lithotomy position. Their findings indicated that hypobaric bupivacaine provided effective anesthesia with a broader yet controllable distribution, resulting in quicker block regression without compromising hemodynamic stability. This aligns with our observations and suggests that hypobaric bupivacaine could be an optimal choice for anorectal surgeries requiring efficient turnover and minimal postoperative monitoring.^[12,13]^

These findings suggest that hypobaric bupivacaine may offer practical benefits in day-case anorectal surgery by facilitating faster patient turnover without compromising patient safety or hemodynamic stability. This recovery profile aligns with the goals of ambulatory surgical care, supporting hypobaric bupivacaine as an effective choice for procedures requiring timely discharge.

Both groups demonstrated similar levels of hemodynamic stability, postoperative pain control, and patient satisfaction, indicating that both anesthetic techniques were effective and safe. However, faster discharge times associated with hypobaric bupivacaine could lead to increased patient turnover and reduced healthcare costs.

The use of the FastTrack criteria ensured that all patients met the necessary conditions for safe discharge, including stable vital signs, effective pain management, recovery of motor function, ability to intake without nausea, adequate hydration status, and normal bladder function. This is consistent with previous studies that emphasized the importance of comprehensive criteria for patient discharge after day-case surgery.^[3,7–10]^

The shorter sensory block duration in the hypobaric bupivacaine group is advantageous for day-case procedures as it allows for quicker recovery and discharge. This aligns with previous research highlighting the benefits of low-dose hypobaric bupivacaine for spinal anesthesia in ambulatory surgery settings.^[4]^

The safety profiles of both hyperbaric and hypobaric bupivacaine were comparable, with no significant differences in the incidence of adverse effects, such as bradycardia, hypotension, nausea, vomiting, agitation, shivering, or respiratory depression. This reinforces the feasibility of using hypobaric bupivacaine as a standard practice in day-case anorectal surgeries and provides a reliable alternative to hyperbaric bupivacaine.^[5]^

The effectiveness and safety of hypobaric bupivacaine in day-case anorectal surgeries were further supported by comparing hyperbaric and isobaric bupivacaine. The benefits of hypobaric bupivacaine, including faster discharge and minimal complications, make it a suitable choice for such procedures.^[4]^

This finding aligns with recent literature, such as the study by Imbelloni et al,^[12]^ which reported that hypobaric bupivacaine provides effective anesthesia with minimal hemodynamic fluctuations, rendering it suitable for outpatient procedures.

A recent systematic review and meta-analysis also highlighted the advantages of using hypobaric bupivacaine for lower-body surgeries, showing improved outcomes in terms of the onset and duration of anesthesia.^[5]^

### 5.1. Implications for clinical practice

The findings of this study have significant implications for clinical practice. Hypobaric bupivacaine can enhance the efficiency of daytime anorectal surgery by reducing the time required for recovery and discharge. This can lead to better resource utilization in healthcare settings and improved patient satisfaction owing to shorter hospital stays and shorter recovery times.^[1]^

Additionally, the safety profiles of hyperbaric and hypobaric bupivacaine were comparable with no significant adverse events reported in either group. This reinforces the feasibility of using hypobaric bupivacaine as a standard practice in day-case anorectal surgeries and provides a reliable alternative to hyperbaric bupivacaine.^[3]^

### 5.2. Future research directions

Although this study provides valuable insights into the use of hypobaric versus hyperbaric bupivacaine in day-case anorectal surgery, further research is warranted to explore the long-term outcomes, patient-reported satisfaction, and cost-effectiveness of these anesthetic techniques in larger multicenter trials. Additionally, investigating the use of other local anesthetics and combinations of regional anesthesia techniques could provide more comprehensive guidance for optimizing anesthesia care in day-case surgical settings.^[5]^

### 5.3. Declaration of generative AI and AI-assisted technologies in the writing process

During the preparation of this work, the author(s) used Open to check for grammatical and spelling errors. After using this tool/service, the author(s) reviewed and edited the content as required and took full responsibility for the content of the published article.

## Author contributions

**Conceptualization:** Semih Baskan.

**Data curation:** Kudret Yasemin Yalniz.

**Formal analysis:** Semih Baskan.

**Investigation:** Kudret Yasemin Yalniz.

**Methodology:** Kudret Yasemin Yalniz.

**Project administration:** Semih Baskan.

**Supervision:** Semih Baskan.

**Validation:** Semih Baskan.

**Writing – original draft:** Semih Baskan.

**Writing – review & editing:** Kudret Yasemin Yalniz, Semih Baskan.
